# Just the Facts: What are the roles of oxygen escalation and noninvasive ventilation in COVID-19?

**DOI:** 10.1017/cem.2020.396

**Published:** 2020-05-13

**Authors:** Brit Long, Stephen Y. Liang, Christopher Hicks, Michael Gottlieb

**Affiliations:** * Brooke Army Medical Center, Department of Emergency Medicine, Fort Sam Houston, TX; † Divisions of Emergency Medicine and Infectious Diseases, Washington University School of Medicine, St. Louis, MO; ‡ Division of Emergency Medicine, Department of Medicine, University of Toronto, Toronto, ON; § Department of Emergency Medicine, Rush University Medical Center, Chicago, IL

**Keywords:** Airway, COVID-19, critical care, emergency medicine

## Abstract

A 37-year-old female presents with cough, fever, dyspnea, and myalgias for five days after recent contact with a family member with confirmed 2019 coronavirus disease (COVID-19). Her vital signs include T 38.3° C, HR 108, BP 118/70 mm Hg, RR 26 breaths per minute, and oxygen saturation 67% on room air. She is not in respiratory distress currently and is protecting her airway. Her chest X-ray reveals bilateral airspace opacities. You plan to immediately intervene and address her hypoxia.

## CLINICAL SCENARIO

A 37-year-old female presents with cough, fever, dyspnea, and myalgias for five days after recent contact with a family member with confirmed 2019 coronavirus disease (COVID-19). Her vital signs include T 38.3° C, HR 108, BP 118/70 mm Hg, RR 26 breaths per minute, and oxygen saturation 67% on room air. She is not in respiratory distress currently and is protecting her airway. Her chest X-ray reveals bilateral airspace opacities. You plan to immediately intervene and address her hypoxia.

## KEY CLINICAL QUESTIONS

1.: **How can oxygen therapy be escalated to avoid intubation?**

Escalation of oxygen therapy includes a stepwise progression of devices meant to improve oxygenation and reduce patient work of breathing.^1–3^ While this escalation may vary based on institution and local infection prevention policies, the basic strategy includes the following:

1. Nasal cannula at 6 liters per minute covered with a surgical mask
2. Venturi mask (which allows for more precise oxygen titration) up to 50% covered with a surgical mask, or non-rebreather if a Venturi mask is not available
3. Nasal cannula at 6 liters per minute plus non-rebreather mask up to 15 liters per minute covered with a surgical mask
4. High-flow nasal cannula covered with a surgical mask
5. Continuous positive airway pressure (CPAP) with viral filter
6. Endotracheal intubation

At each step in a progression, the patient should be closely monitored, including respiratory status (e.g., work of breathing, respiratory rate) and oxygen saturation.^2,3^ If these do not improve, patient repositioning and/or progression is recommended. Patients with increased work of breathing and hypoxemia due to COVID-19 who fail escalation of oxygen therapy and demonstrate clinical distress should undergo endotracheal intubation.^2^

Wherever possible, patients with respiratory distress or hypoxemia and suspected or confirmed COVID-19 should first be placed in a negative pressure room or private room with the door closed given the potential need for aerosol-generating procedures. Providers managing these patients should don appropriate personal protective equipment (PPE) compliant with airborne, contact, and standard precautions with the addition of eye protection.^4^

2.: **What is high flow nasal cannula, and how should it be started?**

High-flow nasal cannula therapy is a system that delivers heated (37° C) and humidified (100% relative humidity) oxygen at concentrations ranging from 21%–100% at flow rates reaching 60 liters per minute.^2,3^ Flow rate and the fraction of inspired oxygen (FiO2) can be titrated based on patient requirements, reducing anatomical dead space, providing positive pressure, reducing inspiratory effort, and improving dynamic compliance.^1,2^ High-flow nasal cannula is also more comfortable than other noninvasive positive pressure ventilation (NIPPV) devices.^2^ This system has demonstrated utility in the management of patients with respiratory distress and COVID-19, with guidelines recommending its use as the first-line strategy for patients with acute hypoxemic respiratory failure, despite conventional oxygen therapy.^1,2,5^ Even though high-flow nasal cannula provides flow rates at 40–60 liters per minute, a cough has flow rates approaching 400 liters per minute, and evidence suggests that high-flow nasal cannula is not associated with high risk of viral transmission when appropriately connected.^6,7^ If used, the FiO2 should be set at 100% with an initial flow rate set to 20 liters per minute. This can be titrated to patient comfort and an oxygen saturation of over 88%. A surgical mask should be placed over the high-flow nasal cannula device to further reduce the risk of viral transmission.^2, 8^

3.: **What is the role of noninvasive positive pressure ventilation (NIPPV) measures for the patient with suspected or confirmed COVID-19?**

NIPPV other than high-flow nasal cannula may have a role in the management of hypoxemic COVID-19 patients.^1–3^ Loss of lung perfusion regulation, hypoxic vasoconstriction, and atelectasis are potential causes of hypoxemia in these patients.^2,9^ CPAP can reduce work of breathing and improve oxygenation, while bilevel positive airway pressure (BiPAP) generally improves ventilation and can improve tidal volumes.^2,9^

If NIPPV is to be used, CPAP is recommended over BiPAP, as some patients with COVID-19 may have normal lung compliance early in the course of the disease.^2,9^ BiPAP can be used in those with obstructive airway disease. CPAP should be considered in patients who have failed high-flow nasal cannula or if high-flow nasal cannula is not available, those who prefer to not be intubated, in centers where there are no mechanical ventilators, and as a device to improve oxygenation during the apneic period prior to intubation.^1,2^ For the patient with respiratory distress, positive end expiratory pressure should be started at 5 mm Hg and titrated based on patient comfort. Providers should monitor patient mental status, oxygenation, and ventilation efficacy (e.g., tidal volume on the CPAP device).^1–3^

4.: **What is the risk to providers of using NIPPV, and how can this risk be minimized?**

NIPPV is thought to be an aerosol-generating procedure that may increase the risk of disease transmission.^1,2,4^ If available, NIPPV should be used in a negative pressure room, and all providers entering the room should use airborne precautions with appropriate PPE.^1,4^ A viral filter should be used on the NIPPV mask before other devices, and an appropriate seal on the patient's mask is essential to reducing disease transmission.^2,3^ A helmet interface can reduce the risk of disease transmission and reduce the risk of aspiration. However, the helmet interface is not available at many institutions.^2^ Cohorting patients with COVID-19 who require NIPPV is also helpful in reducing transmission to providers and other patients.

5.: **What is the role of awake proning or patient repositioning, and how can it be performed?**

Recent evidence suggests that awake proning in patients with COVID-19 improves oxygen saturation compared with supine position, and the authors have found patient repositioning to improve respiratory status and oxygenation in patients with normal mental status who are able to move themselves.^10^ The patient should change position every 30–120 minutes (e.g., prone, right lateral recumbent, left lateral recumbent, sitting upright at 60–90 degrees). Prone position can also be used but is difficult in patients on NIPPV. During and immediately after each position change, the oxygen connection should be assessed. Ten to 15 minutes after the position change, the provider should evaluate patient respiratory status and oxygen saturation. If either has worsened, the patient should attempt a different position. If respiratory status and oxygen saturation continue to decline, progression in oxygen therapy is recommended as described previously.

**KEY POINTS**

• If possible, patients with respiratory distress or hypoxemia should be placed in a negative pressure room or private room with the door closed given the potential need for aerosol-generating procedures. Providers managing these patients should wear appropriate PPE.
• A stepwise progression of oxygen therapy is recommended: 1) Nasal cannula at 6 liters per minute, 2) Venturi mask up to 50% or non-rebreather mask, 3) Nasal cannula plus non-rebreather mask, 4) High-flow nasal cannula, 5) CPAP, and 6) endotracheal intubation.
• High-flow nasal cannula has demonstrated utility in reducing work of breathing and improving oxygenation. If available, it is recommended before the use of other NIPPV devices.
• For patients who fail high-flow nasal cannulaor if high-flow nasal cannulais not available, NIPPV should be considered.
• NIPPV devices are aerosol-generating procedures and may increase disease transmission.
• Awake patient repositioning may assist with lung recruitment and improve oxygen saturation.

## CASE RESOLUTION

You place the patient on a nasal cannula, but her saturation does not increase above 70%. You escalate oxygen therapy while using awake repositioning, and, finally with high-flow nasal cannula, the patient's work of breathing improves and oxygen saturation reaches 90%.

**Figure 1.**
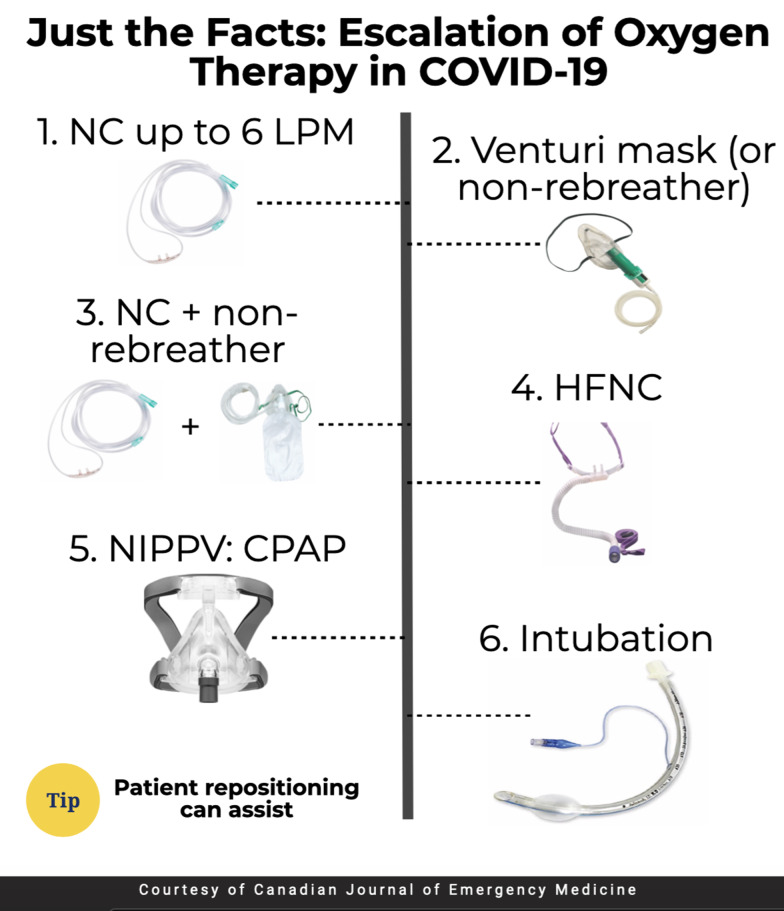
Oxygen escalation therapy. CPAP = Continuous positive airway pressure; HFNC = High-flow nasal cannula; NC = Nasal cannula; NIVVP = noninvasive positive pressure ventilation.

## OTHER RESOURCES

1. https://rebelem.com/covid-19-hypoxemia-a-better-and-still-safe-way/
2. https://emcrit.org/emcrit/avoiding-intubation-and-initial-ventilation-of-covid19-patients/.
3. https://emcrit.org/emcrit/covid-airway-management/.
4. https://emcrit.org/emcrit/covid-respiratory-management/.
5. https://emcrit.org/emcrit/awake-pronation/.
